# Chronic Kidney Disease and Dietary Supplementation: Effects on Inflammation and Oxidative Stress

**DOI:** 10.3390/vetsci8110277

**Published:** 2021-11-15

**Authors:** Elisa Martello, Francesca Perondi, Natascia Bruni, Donal Bisanzio, Giorgia Meineri, Ilaria Lippi

**Affiliations:** 1 Division of Epidemiology and Public Health, School of Medicine, University of Nottingham, Nottingham NG5 1PB, UK; 2 Department of Veterinary Science, University of Pisa, San Piero a Grado (PI), 56124 Pisa, Italy; f.perondi87@gmail.com (F.P.); ilaria.lippi@unipi.it (I.L.); 3 Candioli Pharma S.r.l., S.da Antica di None 1, 10092 Beinasco, Italy; natascia.bruni@candioli.it; 4 RTI International, Washington, DC 20005, USA; dbisanzio@rti.org; 5 Department of Veterinary Sciences, University of Turin, Largo Paolo Braccini 2, Grugliasco, 10095 Torino, Italy; giorgia.meineri@unito.it

**Keywords:** CKD, dog, diet supplement, serum phosphorus, SDMA, ROS

## Abstract

Chronic kidney disease (CKD) causes an irreversible loss of kidney functioning in dogs. This double-blind case-control study evaluates the efficacy of a dietary supplement, which contains calcium carbonate, calcium lactate-gluconate, chitosan, sodium bicarbonate, Lactobacillus acidophilus D2/CSL, *Olea europaea* L. extract, and fructooligosaccharides, in dogs in advanced CKD stage. Thirty dogs were enrolled in the study; half were administered the new dietary supplementation for 90 days, while the others were used as controls. Hematologic, biochemical, and urinalysis were performed. This novel dietary supplement mainly reported a good control of uremia, phosphate, acid-base balance, blood pressure, inflammation, and oxidative stress in dogs with advanced stages of CKD.

## 1. Introduction

Chronic Kidney Disease (CKD) causes an irreversible loss of kidney functions. It is typically considered a disease of older animals, but it can occur at any age. The severity of this disease is classified into different stages (1–4) according to the International Renal Interest Society (IRIS) Guidelines [1]. Even if no cure is possible, veterinarians attempt to manage the disease, suggesting diet modifications in addition to traditional drugs or alternative therapies.

Research shows that restricting protein intake using a balanced renal diet can slow the progression of renal damage and reduce clinical signs (vomiting, lethargy, anorexia, diarrhea, oral ulcerations, polydipsia, polyuria, anemia) mainly caused by an accumulation of the breakdown of products of protein metabolism [2]. Given the frequent hypertension characterizing these individuals, sodium restriction in these diets is also helpful to control blood pressure, minimizing the risk of extra-renal target organ damage [2]. Unfortunately, in most cases, diet manipulation alone is not sufficient at controlling the rise in blood pressure, and hypertension is mainly treated with an angiotensin converting enzyme inhibitor (ACEI, such as benazepril, [1]). Alternatively, research shows that the use of *Olea europaea* could work as an anti-hypertensive agent [3].

Particular attention should be paid to mineral balance. One of the first alterations in patients is the retention of phosphorus (P) by the damaged kidneys [4] and its increased level in the blood (hyperphosphatemia). Indeed, one of the objectives of CKD management is the normalization of the P level by using phosphate binders (i.e., aluminum salts, calcium carbonate, calcium acetate, chitosan, calcium lactate-gluconate) that are able to reduce the absorption of P and calcium (Ca) in the intestine [5,6,7,8,9]. Apart from the alteration of P metabolism, Ca level has to be carefully monitored because it varies by subject and its concentration may increase, decrease, or remain normal [10]. Particular attention should be given to the administration of calcium containing enteric phosphate binders, which could lead to an increase in total and ionized calcium concentrations.

The progression of CKD can cause acid-base abnormalities resulting from the alteration of normal kidney functioning to correctly excrete hydrogen ions and retain bicarbonate ions. This can lead to a metabolic disorder called metabolic acidosis. For limiting such a problem, the use of alkalinizing agents (oral sodium bicarbonate or potassium citrate) is a common choice for veterinarians [1,6,8,11,12].

Individuals with CKD also experience an alteration of the microbiota, indicating an increase in proteolytic bacteria (i.e., Clostridium) and a decrease in beneficial bacteria (i.e., Lactobacilli) [7,13,14].

In addition, inflammation and oxidative stress is common in patients with CKD and can lead to a worsening of clinical symptoms [15,16,17]. For all of these reasons, the choice of probiotics (e.g., Lactobacilli spp. and Bifidobacteria), prebiotics (e.g., fructooligosaccharides and galactooligosaccharides), and antioxidants (e.g., vitamin E, carotenoids, lutein, vegetable oil) has increased in both human and veterinary medicine [18,19,20,21,22,23].

In fact, beyond the use of diets specifically formulated for animals affected by renal diseases, and an increase in water intake, research shows that the addition of supplements could be a valuable alternative to pharmacological therapy in dogs with CKD [8,12,22]. Diet alone is not sufficient at controlling the disease, especially in advanced CKD stages [11].

As described previously, animals with CKD are affected by multiple alterations (regarding their health statuses). The search for a therapy that is able to control all of these alterations, with minimal side effects, and one that is easy to administer, would be a beneficial approach toward managing the disease. Thus, in this double-blind case-control trial, we examined the synergic effects of several natural ingredients in dogs with CKD. The supplement under study (Renal Combi, Candioli, Beinasco, Italy) contained calcium carbonate, calcium lactate-gluconate, chitosan, sodium bicarbonate, *Lactobacillus acidophilus* D2/CSL, *Olea europaea* L. extract, and fructooligosaccharides.

## 2. Materials and Methods

### 2.1. Study Design

A double-blind case-control study on dogs affected by CKD was performed. Diagnosis of CKD based on persistent azotemia, typical clinical findings (such as progressive weight loss, poor appetite and/or polyuria-polydipsia (PU/PD), and renal chronic structural abnormalities as “ultra-soundly” confirmed. Staging of CKD was assigned according to the IRIS [1]. All dogs affected by other concomitant diseases (pre-renal or post-renal azotemia, acute kidney injury, genitourinary tract inflammation or infection, urinary tract obstruction, neoplasia, hypothyroidism, chronic heart failure, and diabetes) were excluded from the study. The study duration was set at 90 days.

### 2.2. Animals

A total of 30 dogs were included in the trial that were randomly assigned to a treated (TRT, n = 15) and a control (CTR, n = 15) group. Both investigator and owner did not know this assignment until the end of the study. Owners were aware of the study protocol and signed an informed consent. The experimental protocol was designed according to the guidelines of the current European and Italian laws on the protection of animals used for scientific purposes (directive 2010/63/EU, put into force in Italy, D.L. 2014/26). Furthermore, the experimental protocol was approved by the Ethical Committee of the Department of Veterinary Sciences of the University of Turin (Italy) (Prot.n.708, 17/03/2021).

### 2.3. Veterinary Evaluations

A complete clinical examination, blood, and urinary test analyses were performed at the beginning of the study (T0), at days 30 (T30), 60 (T60), and 90 (T90).

Clinical examination included simultaneous measurement of body weight (BW; pet scale, four sensors, maximum 100 kg, d¼100 g; Momert, Dunaujvaros, Papírgyáriút 12, 2400, Hungary), and body condition score (BCS; 1 to 5 scoring system) performed by the same veterinary clinician.

Systolic blood pressure (SBP) and diastolic blood pressure (DBP) were measured (electronic sphygmomanometers-CONTEC) five times and the mean value were recorded.

Results from blood test with complete blood count (CBC, Animal blood counter, scil Vet abc^TM^) included the evaluation of hematocrit (HCT), hemoglobin (HG), red blood cells (RBC), white blood cells (WBC), neutrophil (N), eosinophil (EO), basophil (B), lymphocytes (LYM), and platelets (PLTS).

Serum biochemical profile (Automatic Analyzer-Echo, Edif) included the evaluation of blood urea nitrogen (BUN), creatinine (CREA), phosphorus (P), total protein (TP), albumin (ALB), albumin/globulin ratio A/G), glucose (GLU), alanine aminotransferase (ALT), aspartate aminotransferase (AST), alkaline phosphatase (ALP), bilirubin (BIL), cholesterol (CHOL), symmetric dimethylarginine (SDMA).

Venous blood gas analysis (Abaxis VetScan i-Stat1) was also performed to assess bicarbonate (HCO_3_) and ionized calcium (iCa).

Urine sample collection and urinalysis procedures were performed as previously described by Biasibetti and colleagues [24]. Specifically, urine protein (UP) and urinary specific gravity (USG) were evaluated using the pyrogallol red method and the Jaffè method, respectively. Then, urine protein-to-creatinine ratio (UPC) was calculated.

Inflammatory and oxidative stress parameters were also assessed. In particular, C-reactive protein (CRP, ELISA test for dogs, BD Biosciences, BioTek Instruments, Winooski, VT, USA) and d-ROMs (Free Carpe Diem, Diacron International srl, Grosseto, Italy) levels were determined.

### 2.4. Supplement

The dogs were fed a renal commercial diet to avoid any effect of different diet compositions on the estimated parameters (Royal Canin Renal Canine, 16.0% protein, 0.2% phosphorous, 4090.0 Kcal) and they started feeding with the diet seven weeks before entering in the study. Each dog was fed the right amount, following its metabolic requirement according to the FEDIAF Nutritional Guidelines (Fédération européenne de l’industrie des aliments pour animaux familiers) [25]. The amount of food intake was based on the resting energy requirement (RER) multiplied by an appropriate factor to estimate maintenance energy requirement (MER), as reported here:

RER (kcal/day) = 70 × (BWkg)^0.75^

MER = RER × 1.4/1.5

Dogs in the TRT group were fed the commercial diet with the addition of a new supplement (Renal Combi, Candioli, Italy). The supplement contains calcium carbonate, calcium lactate-gluconate, chitosan, sodium bicarbonate, *Lactobacillus acidophilus* D2/CSL, *Olea europaea* L. extract, and fructooligosaccharides (Table 1). Calcium carbonate and calcium lactate-gluconate bind dietary phosphorus in the intestine [24]. Chitosan has absorption properties (reducing intestinal absorption of phosphorus and nitrogen toxins) and antioxidant effects (free radical scavenging activities) [26]. Sodium bicarbonate is an alkalinizing agent acting against metabolic acidosis and rising serum bicarbonate concentration, serum pH, and the partial pressure of carbon dioxide [27]. *L. acidophilus* and the fructooligosaccharides improve intestinal function and reduce the production of nitrogen catabolites through the modulation of gut microbiota [28]. *Olea europaea* L. extract counteracts oxidative stress and reduces blood pressure [29].

The CTR group was fed the same commercial diet with the addition of a placebo (Table 1). The supplement, as well as the placebo, was provided with feed twice daily at the level of 0.2 kg/kg of body weight.

The owners were asked to record any adverse event during the trial (vomiting, diarrhea, loss of appetite).

Dogs with persistent proteinuria or with a history of hypertension were medically treated as per normal disease course. The therapy was set by the veterinarian as recommended by the IRIS staging system [1] and the dogs continued to take part in the trial.

### 2.5. Statistical Analysis

The effect of the supplement on the parameters recorded during the study was tested using a regression model. The model was built as a generalized linear mixed model (GLMM) with Gaussian likelihood. The model included a non-linear variable describing the link between each sampling time within and between the CTR and TRT group. The identification of the subject was included in the model as a random effect in order to account for repeated measurements and the heterogeneity of individuals. The statistical analysis was performed using the R [30].

## 3. Results

All the 30 dogs included in the study completed the trial. In both TRT and CTR groups, 13 dogs were in IRIS stage 3 and two dogs were in IRIS stage 2. All dogs included remained in the same CKD stage for the entire duration of the trial.

The median age of the dogs was 8.8 (from 6 to 13) and 8.5 (from 5 to 13) years in the TRT and CTR group, respectively. In the TRT group, six dogs were males (two neutered) and nine females (one neutered), while in the CTR group—eight dogs were males and seven females (one neutered). Several dog breeds were represented in the set of animals included in the study (American Staffordshire, bulldog, Bernese mountain dog, bull terrier, border collie, beagle, Labrador, Dalmatian, Doberman, fox terrier, German Shepherd, golden retriever, and poodle).

During the study period the BW and BCS did not differ significantly between and within groups at any time, apart from a significant increase in BCS at T90 compare to the T0 in the TRT group and at T90 in the TRT compared to the CTR group (Table 2).

A significant increase in blood pressure was recorded in the CTR group at T90 for both DBP and SBP and at T60 for SBP, only. A significantly higher SBP was also recorded in the CTR group compared to the TRT group at T90 (Table 3). The blood pressure values were within the normal range in the TRT group during the study, while slightly higher in the CTR group at T60 and T90 for the SBP.

Hematological parameters were recorded at each time point and no significant differences were found between and within groups for the entire period. The values were within the normal range during the entire study. Most of the biochemical parameters remained stable during the trial in both groups. CREA and BUN were higher than the normal range in both groups during the entire trial and no significant difference between groups was found at the beginning of the study. A progressive decrease in CREA was reported in the TRT group; CREA mean value was significantly decreased at T90 compared to T0 and at the same time compared to the CTR group (Table 4). In the CTR group, BUN progressively increased during the study and a significant difference was found at T90. Conversely, this parameter progressively decreased (even not significantly) in the TRT group and it was significantly different from the CTR group at T90. Phosphorous (P) remained stable in the CTR group, while it progressively and significantly decreased in the TRT group. TP significantly decreased in the CTR group and was significantly lower when compared to the TRT group at T90; values in both groups were within the normal range at all times. ALB significantly decreased in the CTR group (*p* < 0.05) and it was significantly lower compared to the TRT group at T90 (*p* < 0.05); the values in both groups were within the normal range. The values of ALT, ASP, ALP, and CHOL are also shown in Table 4. SDMA significantly and progressively decreased in the TRT group during the study and a significant difference between the two groups was reported with the TRT group, having a significantly lower value at T90.

The level of serum iCa and HCO_3_ remained within the normal range in both groups during the trial and (non-significantly) increased at T90 compared to T0 in the TRT group only (Table 5).

The urinary parameters were reported in Table 6. The UPC progressively and significantly decreased in the TRT group returning a value within the normal range at T90; the value increased (but not significantly) in the CTR group during the trial remaining higher than the normal range. The two groups differed in UPC value significantly at T60 and T90. The USG decreased in the CTR group and increased in the TRT group but not significantly. Additionally, the two groups differed significantly at T90 when the CTR group value was lower than the TRT group.

The CRP decreased progressively and significantly at T90 in the TRT group; on the other end, the value increased (but not significantly) in the CTR group (Table 7). The two groups also significantly differed in CRP value at T90. The d-ROMs decreased progressively during the trial in the TRT group, while the value increased in the CTR group, but the differences were not significant. The two groups also significantly differed in d-ROMs values at T60 and T90 with the TRT group reporting lower values (Table 7).

No adverse effects (vomiting, diarrhea, anorexia) were reported to the veterinarian by the owners during the study period. The owners found the administration of the supplement easy and no remnants of both supplement/placebo and diet were reported.

## 4. Discussion

In the literature, few studies showed the beneficial effect on the use of dietary supplementation in the CKD progression [12], in the control of hyperphosphatemia and metabolic acidosis in dogs [8] and cats [24]. For example, commercial dietary supplements were tested in dogs affected by CKD and showed a reduction in mortality rate due to uremic crises [12] and a significant improvement in the control of serum phosphate and serum bicarbonate levels [8], without adverse reactions.

In the present investigation, we used a new product (Renal Combi, Candioli S.r.l., Beinasco, Italy) that, in addition to the ingredients listed above, contained *Lactobacillus acidophilus* D2/CSL, *Olea europaea* L. extract, and fructooligosaccharides. Interestingly, the synergic effects of the whole set of ingredients has guaranteed a significant improvement of the renal functioning in the treated animals, even in a shorter period of time, compared to a similar study on dogs (90 vs. 180 days) [8]. Specifically, the product was able to slow down the progression of CKD, to reduce the nitrogen toxins, to control hyperphosphatemia and metabolic acidosis, to counteract the oxidative stress, and to reduce inflammation.

We used SDMA as an early and reliable biomarker of kidney function and we observed that it was significantly decreased in treated dogs at the end of our study (T90). To our knowledge, only a single study investigated SDMA in non-azotemic cats in order to evaluate the efficacy of a dietary supplement [31]. In this previous study, cats with normal CREA but elevated SDMA (meaning early renal insufficiency), when fed a specific diet supplemented with fish oil, antioxidants, L-carnitine, highly bioavailable protein, and amino acids, showed more stable renal functioning compared to cats fed the owner’s-choice foods [31]. In addition, the use of SDMA as a parameter to monitor the progression of CKD in dogs and cats has the advantage of not being affected by lean body mass, as it can happen to another blood parameter: the CREA [28,32]. Indeed, in our study, the importance of having SDMA evaluation in addition to CREA, was confirmed by the significant increase of BCS we found in treated dogs The change in body mass [33] could affect our interpretation of CREA values even if CREA was found significantly decreased at the end of the study in the TRT group [31].

An improvement of renal function in dogs treated with this supplementary diet was also supported by a significant decrease in proteinuria (UPC) and an increase in urinary specify gravity (USG).

This result could be justified by the presence of *Lactobacillus acidophilus* in the new administered product combined with the fructooligosaccharides that make the probiotic more effective. In particular, the effects of *Lactobacillus acidophilus* D2/CSL (CECT 4529) on fecal parameters and body condition in dogs and cats have been recently studied [18,19]. In addition, a study on dogs with CKD treated with multi-strain probiotics containing *Lactobacillus* showed a significant improvement in Glomerular filtration rate (GFR) and USG and a significant reduction of UPC compared to the control [7]. In particular, Lippi et al. supported the important role of the probiotic supplementation in reducing the progression of CKD in dogs [7] and the relationship between intestinal dysbiosis and kidney injury called the “gut-kidney axis” in human medicine [34,35,36]. Research shows that gut microbiota contributes to the generation of several uremic toxins that may promote kidney damage [35]. The use of probiotics in CKD patients could promote the intestinal removal of uremic toxins [7,35], improve GFR [7], and reduce systemic inflammation and proteinuria [13,36].

The level of serum iCa remained within the normal range in both groups during the trial. This is in agreement with a previous study where an increase in values was only recorded from 120 days after the beginning of the treatment, but it was not the cause of any hypercalcemia [8]. This means that a prolonged administration of our new supplement containing calcium carbonate, calcium lactate-gluconate, could provide similar results. In this study, hypercalcemia was not reported in dogs, this being a relevant achievement of our supplement because hypercalcemia can occur in dogs with advanced CKD when conventionally treated with calcium-containing intestinal phosphate-binding agents (i.e., calcium acetate, calcium carbonate, or calcium citrate).

Individuals with CKD require both alkalizing and phosphorus binding therapy when diagnosed. In our study, the effect of sodium bicarbonate included in the supplement helped increase HCO_3_ concentration at the end of the study in the TRT group, even though the difference with T0 was not significant. Values in both groups were below the normal range. This is quite common in cases of severe metabolic acidosis where higher doses or another bicarbonate supplementation might be necessary in order to record values > 18 mmol/L. On the other hand, phosphorous (P) remained stable, but higher, compared to the normal range in the CTR group, while it progressively and significantly decreased in the TRT group at the point of returning to a value close to normal at the end of the study. Hyperphosphatemia is a severe condition and it could be associated with an increase of serum calcium concentration and/or with a risk to develop secondary renal hyperparathyroidism [9]. In particular, it has been associated with negative prognosis and severe morbidity in dogs with CKD. It is more prevalent in dogs with late-stage kidney disease [9]. Based on these results, the chitosan included in the new supplement seems to have prevented the absorption of P and it can be considered a good candidate for a phosphorus-binding therapy substitute as also reported by Martello et al. [8].

Inflammation and oxidative stress seemed to be reduced in dogs treated with the new dietary supplementation. In particular, the inflammatory parameters, CRP and d-ROMS, were significantly decreased in the TRT group, and when compared to the CRT group at the same time point during the trial.

In human studies, it is reported that oxidative stress generates tissue injury and inflammation directly contributing to the progression of CKD [15,37]. In particular, reactive oxygen species (ROS) is usually formed in renal tissue, but during kidney disease, we can attest to an increasing oxidative phosphorylation and ROS accumulation during compensatory mechanisms (when the remaining nephrons concur to hyperfiltration) [2]. On the other hand, in vivo experiments are shown to reduce the production of ROS [37]. One possible explanation for an increase or a decrease in ROS production documented during CKD is a series of dynamic events that may initially increase ROS production, but cannot be maintained with the progression of the kidney disease [37]. ROS could be considered the cause of increased blood pressure in affected animals [3]. However, the presence of oxidative stress in dogs with CKD has already been reported [16,23,38], but few studies have investigated the role of diet in oxidative stress and inflammation in dogs [2]. Recently, Halfen et al. reported that dogs affected by advanced CKD (stage 3 and 4) fed with a renal diet for 6 months, in combination with support treatments, helps control uremia, acidosis, blood pressure, and total antioxidant capacity [2]. Interestingly, our data seem to confirm the same results, but in a shorter period time (90 days vs. six months).

The antioxidants included in the tested supplement, “*Olea europaea*”, could have contributed to the reduction of hypertension in treated dogs. Olive leaf contains the active substances oleuropein, oleracein, and oleanolic acid, and its anti-hypertensive and cholesterol-lowering actions are well documented [3]. In addition, some studies confirmed the anti-hypertensive effect of olive leaf extract in comparison to Captopril in human patients with stage-1 hypertension [3]. To our knowledge, the positive effects of a dietary supplement on blood pressure without the use of a pharmacological therapy (i.e., ACE inhibitors) has never been reported on before [8,12,24]. These preliminary results are of great interest for clinicians, because in dogs with CKD, it is important to control SBP to slow the progression of the disease [1].

Beside these relevant results, some limitations should be noted. The small sample size and the presence of dogs, mostly in stage 3, should be improved, by performing a larger study that includes a representative population of dogs in CKD stages 2, 3, and 4. In addition, a longer study could be of interest in order to evaluate the prognosis and mortality rate for uremic crises, especially in dogs in advanced stages. New study protocols, including laboratory tests on fecal parameters, would be beneficial to show the efficacy of the prebiotics and probiotics present in the supplement on the microbiome.

## 5. Conclusions

In conclusion, this new dietary supplementation provides an efficient control of uremia, phosphate, acid-base balance, BCS, blood pressure, inflammation, and oxidative stress in dogs with advanced stages of CKD.

## Figures and Tables

**Table 1:** Composition of the feed supplement and placebo used during the study.

Ingredients	%
**Feed supplement**	
Vitamin B12 1/1000	10
Vitamin E	0.002
Vitamin B6	0.5
Vitamin C	5
Folic acid	0.2
Lactobacillus acidophilus D2/CSL	0.211
*Olea europaea* L.: olive extract	2
Chitosan	8
Sodium bicarbonate	6
Colloidal silica E551b	0.5
Calcium carbonate	26
Calcium lactate-gluconate	16
Fructooligosaccharides (Profeed^®^ Maxflow)	20
Optimizor uranus	0.2
Maltodextrin	5.387
Total	100.000
**Placebo**	
Maltodextrin	95.00
Appetite stimulants	5.00
Total	100.000

**Table 2:** Body weight (BW) and body condition score (BCS).

Parameter	Time	Group	
		CTR Mean (95% CI)	TRT Mean (95% CI)
BW kg	T0	16.47 (15.36; 16.76)	16.53 (16.25; 17.55)
	T30	16.45 (15.34; 16.76)	16.6 (16.3; 17.62)
	T60	16.44 (15.36; 16.74)	16.73 (16.44; 17.75)
	T90	16.43 (15.35; 16.72)	16.81 (16.51; 17.83)
BCS 1–5	T0	2.9 (2.25; 3.43)	2.81 (2.26; 3.48)
	T30	2.9 (2.29; 3.45)	3.26 (2.75; 3.91)
	T60	2.9 (2.22; 3.43)	3.91 (3.38; 4.58)
	T90	2.91 (2.27; 3.44)	4.31 (3.75; 4.95) *^,$^

Mean and 95% confidence interval (95% CI) resulted from the model in the control (CTR) and treated (TRT) group in the study period: T0 (day 0), T30 (day 30), T60 (day 60), and T90 (day 90). * Significant difference from T0 within group (*p* < 0.05); ^$^ significant difference between groups at the same time (*p* < 0.05).

**Table 3:** Systolic (SBP, normal range < 150 mmHg) and diastolic (DBP, normal range < 95 mmHg) blood pressure.

Parameter	Time	Group	
		CTR Mean (95% CI)	TRT Mean (95% CI)
SBP mm Hg	T0	141.59 (136.23; 147.38)	142.58 (136.8; 147.69)
	T30	149.29 (143.9; 154.97)	142.9 (137.28; 148.5)
	T60	152.78 (147.66; 158.29) *	142.21 (136.81; 147.79)
	T90	155.62 (150.13; 161.57) *	142.98 (137.5; 148.21) ^$^
DBP mm Hg	T0	80.73 (77.77; 83.8)	81.21 (78.09; 84.15)
	T30	85.79 (82.9; 89.15)	82.4 (79.15; 85.51)
	T60	86.34 (83.56; 89.45)	83.27 (80.12; 86.16)
	T90	89.52 (86.62; 92.65) *	83.63 (80.63; 86.62) ^$^

Mean and 95% confidence interval (95% CI) resulted from the model in the control (CTR) and treated (TRT) group in the study period: T0 (day 0), T30 (day 30), T60 (day 60) and T90 (day 90). * Significant difference from T0 within group (*p* < 0.05), ^$^ significant difference between groups at the same time (*p* < 0.05).

**Table 4:** Biochemical parameters: blood urea nitrogen (BUN), creatinine (CREA), phosphorus (P), total protein (TP), albumin (ALB), albumin/globulin (AG), glucose (GLU), alanine aminotransferase (ALT), aspartate aminotransferase (AST), alkaline phosphatase (ALP), bilirubin (BIL), cholesterol (CHOL), symmetric dimethylarginine (SDMA).

Parameter	Laboratory Standard Reference Range	Time	Group	
			CTR Mean (95% CI)	TRT Mean (95% CI)
CREA mg/dL	0.5–1.8	T0	3.2 (2.89; 3.51)	3.17 (2.83; 3.46)
		T30	3.28 (3; 3.59)	2.9 (2.57; 3.19)
		T60	3.24 (2.95; 3.53)	2.7 (2.37; 2.98)
		T90	3.22 (2.92; 3.53)	2.4 (2.03; 2.68) *^,$^
BUN mg/dL	15–45	T0	127.8 (115.79; 143.21)	136.37 (122; 148.88)
		T30	140.26 (128.24; 155.85)	132.52 (118.21; 144.91)
		T60	152.55 (140.03; 168.01)	130.01 (115.83; 142.32)
		T90	165.1 (153.08; 181.03) *	126.62 (112.71; 138.95) ^$^
P mg/dL	2.7–5	T0	8.07 (7.27; 8.93)	8.1 (7.23; 8.92)
		T30	8.32 (7.53; 9.17)	7.41 (6.56; 8.23)
		T60	8.55 (7.76; 9.42)	6.97 (6.08; 7.8)
		T90	8.78 (7.98; 9.62)	5.71 (4.86; 6.55) *^,$^
TP mg/dL	6–7.5	T0	5.84 (5.63; 6.02)	5.66 (5.47; 5.89)
		T30	5.64 (5.41; 5.83)	5.72 (5.51; 5.95)
		T60	5.48 (5.25; 5.67)	5.73 (5.53; 5.96)
		T90	5.34 (5.11; 5.52) *	5.82 (5.62; 6.06) ^$^
ALB mg/dL	2.5–4.2	T0	2.09 (1.92; 2.23)	1.87 (1.73; 2.06)
		T30	1.95 (1.79; 2.09)	1.92 (1.78; 2.11)
		T60	1.83 (1.66; 1.98)	2 (1.86; 2.18)
		T90	1.72 (1.55; 1.87) *	2.09 (1.94; 2.29) ^$^
AG	0.5–1.3	T0	0.95 (0.9; 1.03)	0.93 (0.88; 1)
		T30	0.95 (0.9; 1.03)	0.94 (0.89; 1)
		T60	0.92 (0.86; 0.99)	0.93 (0.87; 0.99)
		T90	0.91 (0.83; 0.96)	0.92 (0.84; 0.96)
GLU mg/dL	50–100	T0	87.44 (86.2; 89.06)	87.23 (86; 88.7)
		T30	86.85 (85.33; 88.23)	87.11 (85.75; 88.39)
		T60	86.32 (84.64; 87.48)	86.99 (85.64; 88.3)
		T90	87.84 (86.62; 89.73)	86.87 (85.28; 88.14)
ALT UI/L	7–40	T0	80.43 (79.26; 81.56)	80.66 (79.64; 81.83)
		T30	80.01 (78.77; 81.11)	80.53 (79.49; 81.56)
		T60	80.93 (79.89; 82.22)	80.34 (79.24; 81.33)
		T90	80.42 (79.42; 81.55)	80.2 (79.05; 81.37)
AST UI/L	7–40	T0	50.06 (49.51; 50.57)	50.41 (49.89; 51)
		T30	50.24 (49.72; 50.81)	50.24 (49.71; 50.77)
		T60	50.28 (49.78; 50.9)	50.09 (49.52; 50.61)
		T90	50.2 (49.68; 50.79)	49.87 (49.28; 50.38)
ALP UI/L	5–110	T0	188.03 (185.84; 190.13)	188.93 (186.84; 191.17)
		T30	187.4 (185.21; 189.57)	188.01 (185.9; 190.28)
		T60	187.56 (185.4; 190.05)	187.05 (184.54; 189.3)
		T90	187.26 (185.2; 189.37)	185.55 (182.97; 187.72)
BIL mg/dL	0–0.7	T0	0.28 (0.25; 0.32)	0.27 (0.22; 0.3)
		T30	0.28 (0.25; 0.33)	0.27 (0.24; 0.3)
		T60	0.28 (0.25; 0.32)	0.28 (0.24; 0.31)
		T90	0.29 (0.26; 0.33)	0.28 (0.24; 0.31)
CHOL mg/dL	140–240	T0	388.03 (384.59; 391.78)	388.07 (384.52; 391.68)
		T30	389.49 (385.72; 393.82)	387.47 (383.49; 391.19)
		T60	388.99 (385.47; 393.09)	387.36 (383.42; 390.96)
		T90	385.03 (380.95; 388.62)	387.42 (383.67; 391.08)
SDMA µg/dL	<18	T0	33.97 (30.9; 37.36)	35.09 (31.38; 38.47)
		T30	35.15 (31.92; 38.59)	31.56 (27.9; 34.87)
		T60	34.62 (31.44; 38.2)	28.74 (25.26; 31.85)
		T90	35.34 (32.2; 38.8)	25.58 (22.06; 28.88) *^,$^

Mean and 95% confidence interval (95% CI) resulted from the model in the control (CTR) and treated (TRT) group in the study period: T0 (day 0), T30 (day 30), T60 (day 60), and T90 (day 90). * Significant difference from T0 within group (*p* < 0.05), ^$^ significant difference between groups at the same time (*p* < 0.05).

**Table 5:** Serum bicarbonate (HCO_3_) and ionized calcium (iCa).

Parameter	Laboratory Standard Reference Range	Time	Group	
			CTR Mean (95% CI)	TRT Mean (95% CI)
HCO_3_ mmol/L	18–24	T0	16.61 (15.97; 17.22)	16.51 (15.84; 17.16)
		T90	16.60 (15.96; 17.2)	17.32 (16.66; 17.99)
iCa mmol/L	1.29–1.41	T0	1.34 (1.28; 1.39)	1.32 (1.26; 1.37)
		T90	1.35 (1.29; 1.4)	1.40 (1.34; 1.46)

Mean and 95% confidence interval (95% CI) resulted from the model in the control (CTR) and treated (TRT) group in the study period: T0 (day 0), T30 (day 30), T60 (day 60), and T90 (day 90).

**Table 6:** Urinary parameters: proteinuria (UPC) and urinary specify gravity (USG).

Parameter	Laboratory Standard Reference Range	Time	Group	
			CTR Mean (95% CI)	TRT Mean (95%CI)
UPC	<0.5	T0	0.73 (0.53; 0.94)	0.67 (0.46; 0.87)
		T30	0.8 (0.6; 1.02)	0.62 (0.41; 0.82)
		T60	0.87 (0.67; 1.09)	0.46 (0.25; 0.65) ^$^
		T90	0.95 (0.76; 1.17)	0.24 (0.03; 0.44) *^,$^
USG	-	T0	1014.68 (1009.79; 1019.17)	1015.04 (1010.74; 1019.42)
		T30	1012.71 (1007.95; 1016.89)	1016.25 (1012.07; 1020.6)
		T60	1010.59 (1005.85; 1015.01)	1019.22 (1014.89; 1023.74)
		T90	1007.81 (1003.11; 1012.21)	1023.51 (1019.17; 1027.95) ^$^

Mean and 95% confidence interval (95% CI) resulted from the model in the control (CTR) and treated (TRT) group in the study period: T0 (day 0), T30 (day 30), T60 (day 60), and T90 (day 90). * Significant difference from T0 within group (*p* < 0.05), ^$^ significant difference between groups at the same time (*p* < 0.05).

**Table 7:** C-reactive protein (CRP) and reactive oxygen metabolite-derived compound (d-ROMs).

Parameter	Laboratory Standard Reference Range	Time	Group	
			CTR Mean (95% CI)	TRT Mean (95% CI)
CRP mg/dL	0-0.1	T0	0.65 (0.55; 0.76)	0.81 (0.69; 0.92)
		T30	0.65 (0.55; 0.76)	0.74 (0.63; 0.86)
		T60	0.73 (0.62; 0.84)	0.65 (0.54; 0.76)
		T90	0.82 (0.72; 0.93)	0.56 (0.45; 0.66) *^,$^
d-ROMs U CARR		T0	113.7 (104.83; 124.8)	113.26 (103.08; 121.81)
		T30	119.25 (110.28; 130.18)	106.66 (96.61; 115.35)
		T60	123.53 (114.78; 134.54)	102.85 (93.09; 111.52) ^$^

Mean and 95% confidence interval (95% CI) resulted from the model in the control (CTR) and treated (TRT) group in the study period: T0 (day 0), T30 (day 30), T60 (day 60), and T90 (day 90). * Significant difference from T0 within group (*p* < 0.05), ^$^ significant difference between groups at the same time (*p* < 0.05).

## Data Availability

Data available upon request to the authors.
